# Associations of intermediate hyperglycemia with elevated abdominal obesity, high-sensitivity C-reactive protein, and leptin in Korean adults

**DOI:** 10.7586/jkbns.24.016

**Published:** 2024-08-22

**Authors:** Heashoon Lee

**Affiliations:** Department of Nursing, Hannam University, Daejeon, Korea

**Keywords:** Obesity, abdominal, Blood glucose, Glycated hemoglobin, C-reactive protein, Leptin

## Abstract

**Purpose:**

This study investigated the associations between intermediate hyperglycemia (IH) and increased body mass index (BMI), abdominal obesity (AO), high-sensitivity C-reactive protein (hs-CRP), and leptin levels in Korean adults.

**Methods:**

The participants were 248 adults (≥ 19 years) who understood the purpose of the study, had no cognitive impairment, and were able to communicate. Physical examinations, BMI, AO measurements, and blood tests were performed. Data were analyzed using the t-test, chi-square test, Pearson correlation coefficients, and multiple logistic regression analyses. The risk factors for IH were predicted after adjusting for BMI, waist circumference (WC), age, hs-CRP and leptin levels, education, and economic status.

**Results:**

WC, hs-CRP and leptin levels, and age were higher in the IH group than in the non-IH group. According to the multiple logistic regression analysis, the factors affecting IH prevalence were WC, hs-CRP, leptin, and age. AO (male, WC ≥ 90 cm; female, WC ≥ 85 cm) exhibited an adjusted odds ratio of 5.45 for IH. IH was 2.43 times higher in those with hs-CRP > 3 mg than in those with hs-CRP < 1 mg. As leptin levels increased, the odds ratio for IH increased by 3.05 times. IH was 8.07, 8.79, 18.42, and 35.33 times more common for those in their 30s, 40s, 50s, and ≥ 60 years of age, respectively, than those 19–29 years old.

**Conclusion:**

This study provides evidence that interventions for AO should be included in IH management programs. Identifying the predictive factors for IH may contribute to the early detection of type 2 diabetes mellitus.

## INTRODUCTION

The International Diabetes Federation estimates that the prevalence of diabetes in the global adult population will be 10.5% in 2021 and 12.2% in 2045 [1]; 90%–95% of cases are type 2 diabetes mellitus (T2DM) [2]. Worldwide, approximately 50% of adults with diabetes have not been diagnosed, which can lead to serious vascular complications and premature death [1]. Intermediate hyperglycemia (IH) is a condition in which blood glucose levels do not meet the criteria for diabetes but have abnormal carbohydrate metabolism [3] and impaired glucose regulation [4]. IH prevalence is increasing worldwide [3], if left untreated, 37% of people with IH will develop T2DM within 4 years [5]. Both IH and T2DM are diagnosed based on glucose criteria that measure fasting blood glucose (FBS) and hemoglobin A1c (HbA1c) [6]. As the prevalence of diabetes continues to increase, more sensitive and practical methods to detect IH are needed [1]. As an adverse effect of IH, the risk of developing DM increases due to poor blood sugar control and continuous exposure to hyperglycemia [1,3,5]. IH also has a higher risk of developing cardiovascular disease due to unstable glycemic control and potential changes in blood pressure and lipid levels [7,8].

Obesity is positively correlated with IH and is a major risk factor for exacerbating IH [9]. Obesity is a risk factor that exacerbates inflammatory conditions and increases the incidence of impaired fasting glucose [10,11]. Obesity status is generally assessed using body mass index (BMI); however, the BMI scale has limitations such as its inability to account for variations in body composition [2]. As an indicator of abdominal obesity (AO), waist-to-hip ratio (WHR) may be appropriate for comparisons between different races and genders [7,12,13]. Waist circumference (WC) is a direct measure of the amount of abdominal fat and is useful in assessing AO. It is also a simple and reliable alternative measure of body composition [12]. Based on WC, AO is 90 cm or more for men and 85 cm or more for women, which is particularly useful for measuring the AO of normal or overweight individuals based on BMI [14,15]. AO may occur because of the accumulation of abdominal visceral fat when the BMI is normal [12]. Our study was conducted on Koreans, a single race, and AO was classified based on WC.

AO is also positively associated with IH, and is a major risk factor for IH exacerbation [3]. AO increases IH prevalence because fat tissue induces insulin resistance (IR) and decreases the function of Langerhans cells, which secrete insulin in people with obesity [16]. IR and elevated blood sugar levels are more closely associated with visceral fat than with fat in other areas [14]. Asians accumulate more visceral fat than Westerners, despite having lower BMIs, which leads to a higher risk of IH [17].

Individuals with obesity exhibit a greater abundance of macrophages in their adipose tissues, indicating that both adipocytes and macrophages play significant roles in inflammation during obesity [18]. Even though Asians may be classified as normal weight based on BMI, C-reactive protein (CRP) levels are increased in those with AO as measured by WC [13]. CRP is an inflammatory marker, elevated CRP levels are associated with IH and T2DM [19] Inflammation reduces vascular permeability and alters peripheral hemodynamics, blocking the transport of insulin across tissues and cells and promoting IR [18]. It is a key factor in the pathogenesis and development of diabetes [18,19].

Leptin, which is primarily responsible for regulating body weight by achieving a balance between food intake and energy expenditure, also helps regulate glucose homeostasis and insulin sensitivity [2]. Leptin levels are increased in IH. Additionally, obesity and leptin levels are positively correlated [20]. Secretion of both leptin and insulin is affected by the total amount of fat deposits, including short-term changes in energy balance [21]. Leptin and insulin signaling are important for the regulation of brain energy homeostasis [20]. Individuals with obesity have elevated leptin concentrations and insulin and appear to be resistant to appetite loss [12].

Most previous studies on DM have included patients with diabetes [2,7,15,19], obesity [4,7,9], CRP [13,19], leptin [2,20,21], conversion from IH to T2DM [6], and diabetes prevalence [1,3,11]. IH increases the risk of developing DM [3,5]. IH is also associated with a higher risk of developing unstable cardiovascular disease [8]. Therefore, early detection of IH is important. Asians, such as Koreans, often have AO with a normal BMI [17]. Therefore, it is necessary to determine whether IH significantly increases in AO. Moreover, Asians with IH are more susceptible to developing T2DM than Westerners [3]. Leptin is involved in the regulation of insulin sensitivity, so measuring leptin in IH may help elucidate the mechanisms leading to diabetes. Also, because leptin plays a role in regulating appetite and energy balance, checking leptin levels in IH may predict appetite dysregulation and weight management problems [2]. Therefore, it is important to determine the relationship between leptin and IH. To our knowledge, these studies have not confirmed the relationship between obesity and inflammatory markers, which are factors influencing IH in Asian Korean adults, who have a higher conversion rate to T2DM than Westerners [3].

This study investigated the associations among IH, increased BMI, AO, hs-CRP, and leptin biomarkers in Korean adults. In particular, our study targeted adult patients with IH. Identifying the predictive factors for IH may contribute to the early detection of T2DM.

## METHODS

### 1. Study design

This descriptive correlational study investigated the association of IH with increased AO, hs-CRP, and leptin levels in Korean adults.

### 2. Participants and data collection

Participants were recruited through announcements on the hospital website. They agreed to the purpose of the study and enrolled in the hospital's internal medicine outpatient department. The criteria for selecting participants were adults (≥ 19 years) who understood the purpose of the study, had no cognitive impairment, and were able to communicate. The exclusion criteria were individuals with infectious diseases, DM diagnosis, and taking regular medications. Physical examination, BMI, AO measurement, and blood tests were performed on the final participants.

The number of participants was calculated using G*Power 3.1 [22]; significance criteria α = .05, When setting two comparison groups, medium effect size of .15, and statistical power (1-ß) of .80, the number of participants was calculated to be 225. Considering a dropout rate of 10%, 250 participants were recruited. The final analysis was conducted with 248 people, after excluding two who withdrew from participation during the research process.

### 3. Instruments

#### 1) Classification criteria of IH and non-IH groups

According to the American Diabetes Association, FBS was classified as 100–125 mg/dL for the IH group and < 100 mg/dL for the non-IH group; HbA1c was classified as 5.7%–6.4% for the IH group and < 5.7% for the non-IH group [3].

#### 2) Obesity and AO markers measurement

To measure obesity, height and weight were measured after fasting for more than 8 hours and wearing light clothes, BMI was obtained as body weight divided by height squared (kg/m^2^) [23]. In this study, normal weight (BMI < 23.0 kg/m^2^), overweight (BMI = 23.0-24.9 kg/m^2^), obesity (BMI = 25.0-29.9 kg/m^2^), and high obesity (BMI ≥ 30.0 kg/m^2^) were classified [23]. The WC was measured at the midpoint between the lower ribs and the iliac crest [19]. WC ≥ 90 cm for men and WC ≥ 85 cm for women were applied as the AO standard [15].

#### 3) Blood variable measurement

Blood was collected in a vacuum tube by a trained expert who punctured a vacutainer needle into the vein of the participants who had fasted for > 8 hours. The measurement tools, methods, and procedures were identically applied to all participants.

In this study, the FBS levels were measured using the hexokinase UV method (Hitachi Automatic Analyzer 7600-210, Tokyo, Japan). HbA1c levels were measured using high-performance liquid chromatography (Tosoh G8, Tokyo, Japan). The hs-CRP levels were measured by immunoturbidimetry using an automatic hemocytometer (XN 9000 Sysmex, Kobe, Japan). CRP is an inflammatory marker of acute inflammation, whereas obesity-related inflammation is a low-level chronic inflammation; therefore, in this study, high-sensitivity CRP (hs-CRP) measured in the low range of 0–10 mg/L was used [19]. The Centers for Disease Control and Prevention, assess inflammation risk using the hs-CRP classifications normal (hs-CRP < 1 mg/L), moderate inflammation (hs-CRP = 1–3 mg/L), and high inflammation (hs-CRP > 3 mg/L) [8]. This classification was used in this study. Leptin levels in serum were measured by quantitative enzyme-linked immunosorbent assay using an Quantikine™ Human immunoassay kit (R&D System, Minneapolis, USA) with 0.5 a sensitivity following the package instructions.

### 4. Statistical analysis

General characteristics were analyzed by frequency, percentage, mean, and standard deviation. Differences in general characteristics, BMI, AO markers, hs-CRP, and leptin levels between the IH and non-IH groups were analyzed using the t-test and chi-square test. Correlations among BMI, WC, hs-CRP, and leptin levels were analyzed using Pearson’s correlation coefficient. Multiple logistic regression analysis was necessary to confirm the relative contribution of each predictor variable. A multiple logistic regression analysis was performed to identify the factors influencing IH after adjusting for BMI, WC, hs-CRP, leptin, age, and educational and economic levels. According to the sex-specific AO standards, WC was converted into a binary variable for each sex (male, WC ≥ 90 cm = 1, WC < 90 cm = 0; female, WC ≥ 85 cm = 1, WC < 85 cm = 0) (SPSS 26.0, IBM Corp., Armonk, NY, USA).

### 5. Ethical considerations

The study was conducted after prior approval from the Hannam University Institutional Review Board (IRB no. 2023-02-13-0920). The purpose of the study was explained to the director of a hospital located in Seoul and consent was obtained. The researcher explained the purpose of the study, procedures, and possibility of withdrawal during research participation to the participants, and obtained participant consent forms. The data collection period was from October 2023 to February 2024. The collected data were used only as research data, and the anonymity and confidentiality of personal information were guaranteed.

## RESULTS

### 1. General characteristics of the IH and non-IH groups

This study showed that the IH group was 52.4% and non-IH group was 47.6%. The participants’ mean age was higher in the IH group than in the non-IH group (t = 3.16, *p* = .002).

Among participant's aged 60 or older, the IH group (16.2%) was more included than the non-IH group (5.9%) (χ^2^ = 18.45, *p* < .001). The IH group had more college graduates (88.5%); the non-IH group, had more high school graduates (71.2%) (χ^2^ = 11.64, *p* < .001).

In the IH group, the majority were in the high economic level (44.6%); the majority in the non-IH group were in the medium economic level (55.9%) (χ^2^ = 6.63, *p* = .036). There were no significant differences in sex or family members living together between the two groups.

The mean FBS (t = 6.93, *p* < .001) and HbA1c (t = 11.91, *p* < .001) levels were higher in the IH group than in the non-IH group (Table 1).

### 2. BMI, AO markers, Hs-CRP, and leptin level related characteristics in the IH and non-IH groups

The mean BMI (t = 4.17, *p* = .042) was higher in the IH group than in the non-IH group. In the IH group, the majority were in the high-obesity (≥ 30.0 kg/m^2^) (45.4%); the majority in the non-IH group were in the obesity (25.0~29.9 kg/m^2^) (43.2%) (χ^2^ = 16.63, *p* = .001). The mean WC (t = 5.89, *p* < .001) was higher in the IH group than in the non-IH group. The AO (male, WC ≥ 90 cm; female, WC ≥ 85 cm) in WC was higher in the IH group than in the non-IH group. A significant difference in AO was observed between the two groups (χ^2^ = 28.96, *p* < .001).

The mean hs-CRP level was higher in the IH group than in the non-IH group (t = 3.37, *p* = .001). The most common hs-CRP level was < 1 mg (46.2%), in the IH group and < 1 mg (65.3%) in the non-IH group (χ^2^ = 10.28, *p* = .006). The mean leptin level was higher in the IH group than that in the non-IH group (t = 3.35, *p* = .001) (Table 2).

### 3. Correlation among BMI, WC, Hs-CRP, and leptin

The BMI was significantly positive correlated with WC (r = .79, *p* < .001), hs-CRP (r = .49, *p* < .001) and leptin (r = .55, *p* < .001). WC was significantly positive correlated with hs-CRP (r = .41, *p* < .001) and leptin (r = .37, *p* < .001). Furthermore, hs-CRP levels were positively correlated with leptin levels (r = .46, *p* < .001) (Table 3).

### 4. Predictive variables for increased IH

To identify predictors for IH increase, the variables entered into the logistic regression analysis were those with significant differences between the two groups (Table 1, 2). Additionally, the multiple logistic regression analysis performed confirmed the relative contribution of each predictor variable.

The factors affecting IH incidence were WC, hs-CRP, leptin, and age. The adjusted odds ratio for IH was 5.45 times [95% confidence interval (CI) = 2.09 to 12.44, *p* < .001] higher in patients with AO than in those without AO. IH was 2.43 times (95% CI = 1.02 to 6.79, *p* = .004) higher for hs-CRP > 3 mg than for hs-CRP < 1 mg. As leptin levels increase, the odds ratio of IH increases by 3.05 times (95% CI = 1.15 to 10.09, *p* = .003). IH was 8.07 times (95% CI = 2.31 to 28.14, *p* = .001), 8.79 times (95% CI = 2.46 to 31.42, *p* = .001), 18.42 times (95% CI = 4.97 to 68.26, *p* < .001), and 35.33 times (95% CI = 7.82 to 159.64, *p* < .001) higher for those in their 30s, 40s, 50s, and ≥ 60 years of age, respectively, than those aged 19–29 years (Table 4).

## DISCUSSION

This study investigated the association between IH and increased levels of AO, hs-CRP, and leptin in Korean adults. WC was positively correlated with hs-CRP and leptin levels, and hs-CRP levels were also significantly correlated with leptin. As WC, an AO indicator, increased CRP [19], and leptin levels increased [20].

Our study revealed that WC was notably higher in the IH group, a factor that significantly influenced the IH incidence. This is particularly significant as the pathogenesis of obesity and diabetes involves common pathways with increased IR and proinflammatory patterns [2]. Furthermore, the distribution of adipose tissue, as an independent factor in IR increase regardless of BMI [17], leads to pancreatic beta cells secreting insulin inappropriately to overcome this resistance, which in turn leads to T2DM [11].

In particular, excessive visceral fat accumulation without general obesity may be accompanied by enhanced IR and inflammation [3]. AO increases inflammatory cytokines secreted from adipocytes in abdominal fat, inhibits the action of insulin receptors, increases blood insulin concentration, and worsens IR [12,14,15]. On the other hand, in general obesity, more fat is distributed in tissues such as muscles than abdominal fat. Because abdominal fat secretes relatively less inflammatory cytokines, IR may be lower compared to AO [3,12,14]. Therefore, the authors considered AO to be associated with IH. At similar BMI levels, Asians have higher abdominal and visceral fat levels than Caucasian Europeans [3]. AO is also associated with IH [9,11]. Central obesity, including a large WC or WHR , is a predictor of IH and has been shown to increase IH risk of IH 1.50 times [24]. Increased visceral fat increases CRP levels and IH risk [3], and causes the secretion of adipokines (such as tumor necrosis factor and interleukin-6), that interfere with insulin receptor function, in turn increasing IR and exacerbating IH progression [24]

AO is more strongly associated with IH than general obesity indices, such as BMI, which supports our findings that WC had a greater influence on IH. Higher WC increases c conversion from IH to T2DM [17], Therefore, reducing WC improves glycemic control and prevents IH from progressing to T2DM [25].

In this research, the hs-CRP levels were higher in the IH group, which affected IH incidence. Obesity is associated with increased hs-CRP levels, which correlate with WC in middle-aged Korean adults [26]. The effect of hs-CRP (1–3 mg/L) was higher in the AO group than in the group with obesity [23]. AO is an indicator for the relationship between hs-CRP and AO, leading to a higher IH risk [3].

Visceral fat is associated with an increase in hs-CRP, even if the BMI index is normal, hs-CRP increases in individuals with only AO [26]. AO measures are more closely associated with the risk of IH and T2DM than BMI, and AO is associated with hs-CRP independent of BMI [26]. CRP is the most widely measured marker of inflammation, and inter-individual differences are primarily due to genetic factors. However, adiposity is another major determinant of CRP synthesis in the liver through the production of proinflammatory cytokines [19].

After classifying hs-CRP levels into three grades, there was a relationship between the inflammatory factor hs-CRP and IH [27]. Elevated serum CRP levels in middle-aged Asians are associated with increased IH risk factors, especially high HbA1c levels [19]. CRP is associated with IR [23], in addition, when the CRP concentration is high, the risk of progression to diabetes increases [27].

In contrast, lower CRP levels increase the likelihood of blood sugar levels returning to normal. In adult participants, 65.9% returned to normal blood sugar levels during a 4-year follow-up study [27]. These results suggest that hs-CRP level may be a common indicator for verifying IH progression.

Throughout this study, leptin levels, which is a factor that affects IH, were higher in the IH group. Leptin is a hormone secreted by the adipose tissue [21]. The inflammatory response caused by AO causes leptin resistance by interfering with the operation of leptin receptors. Leptin resistance indicates changes in lipid metabolism, which reduces the function of insulin receptors and interferes with the insulin signaling pathway, resulting in IR [2,21]. Leptin increases FBS and HbA1c [21], and is associated with IH [20]. Since leptin affects the development of IH by suppressing insulin secretion [2], individuals with elevated leptin levels had a higher IH rate (20%) than those in the normal group (2%) [20]. Our multiple logistic regression analysis showed that leptin levels increased with IH [20]. An association has been reported between leptin and IR [28]. IR is a key factor in the pathogenesis of IH and T2DM [21].

In the hypothalamus, leptin interacts with leptin receptors to stimulate satiety. Insulin is responsible for maintaining proper energy storage and utilization, whereas leptin reduces the ongoing energy intake. Therefore, both are necessary for the regulation of energy expenditure and glucose homeostasis [29]. Hyperleptinemia has been linked to the presence of IR, however, leptin levels have been negatively correlated with insulin sensitivity [21]. Our study also found that increased leptin levels were associated with IH risk factors.

In our current study, the IH group had older participants, which was a factor affecting IH incidence. In one study, IH increased by 1.30 times in those aged ≥ 65 years than in those aged 50–59 years [11]. The American Diabetes Association that the prevalence of IH in older adults is more than 50% [3], which is similar to that in the Korean adult in this study. As aging progresses, AO increases, and insulin sensitivity decreases owing to a decrease in skeletal muscle mass [12]. Additionally, T2DM prevalence in Korea is higher for those in their 30s (16.7%) and for those aged ≥ 65 years (30.1%), and increases with age [15]. Physiological changes due to aging make blood sugar control difficult, and complications are more likely to occur in older than in younger adults [11]. Since IH is closely related to T2DM, factors associated with IH in older adults must be identified to prevent T2DM [3].

Pathological dysregulation that eventually leads to diabetes is preceded by IH [3]. We believe this is an initial step and opportunity for preventive interventions. Approximately 25% of patients with IH progress to T2DM within 3–5 years, and up to 70% of patients with IH develop diabetes during their lifetime [3].

Our study provides insights for the early detection and prevention of diabetes progression in patients with IH. Reducing AO in patients with IH can prevent the progression to diabetes. AO management is an important intervention for preventing the transition from IH to T2DM [25]. In particular, aerobic exercises, such as walking, running, and cycling, should be implemented to reduce AO [30].

A limitation of this study is that patients could not be excluded who had with liver failure, a factor that interferes with CRP production [19]. Additionally, unmeasured factors such as the amount of exercise, eating habits, and body fat percentage may have influenced the study results. In future studies, it is necessary to identify a more accurate indicator of the amount of abdominal fat by including not only WC in the measurement of AO, but also WHR, which reflects hip circumference.

## CONCLUSION

The results of this study indicated WC, hs-CRP, leptin, and age were factors that affected IH incidence. IH prevention and management programs for Korean adults should include management of AO, hs-CRP, and leptin. This can prevent the conversion to diabetes.

In the present study, patients with IH were able to control their blood sugar levels by monitoring their inflammation levels. This suggests that hs-CRP and leptin may be additional biomarkers for identifying individuals at a high risk of IH. This has clinical significance for the treatment and prevention of IH [27]. Patients should be educated about the potential consequences of poorly managed IH and obesity. Lifestyle modifications that influence leptin levels should be encouraged as proactive measures.

## Figures and Tables

**Table 1.** General Characteristics of the IH and Non-IH Groups (N = 248)

Variables	Categories	Total	IH group	Non-IH group	t/χ² (*p*)
			FBS:100-125 mg/dL or HbA1c: 5.7%–6.4%	FBS < 100 mg/dL or HbA1c < 5.7%	
			n = 130 (52.4%)	n = 118 (47.6%)	
Age (yr)		42.42 ± 12.06	44.69 ± 11.63	39.92 ± 12.09	3.16 (.002)
	19~29	32 (12.9)	7 (5.4)	25 (21.2)	18.45 (< .001)
	30~39	85 (34.3)	46 (35.4)	39 (33.1)	
	40~49	63 (25.4)	36 (27.6)	27 (22.9)	
	50~59	40 (16.1)	20 (15.4)	20 (16.9)	
	≥ 60	28 (11.3)	21 (16.2)	7 (5.9)	
Sex					1.58 (.208)
	Male	126 (50.8)	71 (54.6)	55 (46.6)	
	Female	122 (49.2)	59 (45.4)	63 (53.4)	
Family living together					0.88 (.347)
	Yes	191 (77.0)	104 (80.0)	87 (73.7)	
	Alone	57 (23.0)	26 (20.0)	31 (26.3)	
Education level					11.64 (< .001)
	High school	49 (19.8)	15 (11.5)	34 (28.8)	
	Above college	199 (80.2)	115 (88.5)	84 (71.2)	
Economic level					6.63 (.036)
	Low	34 (13.7)	16 (12.3)	18 (15.3)	
	Medium	122 (49.2)	56 (43.1)	66 (55.9)	
	High	92 (37.1)	58 (44.6)	34 (28.8)	
FBS (mg/dL)		98.98 ± 27.52	109.56 ± 34.28	87.31 ± 6.48	6.93 (< .001)
HbA1c (%)		5.66 ± 0.43	5.91 ± 0.44	5.39 ± 0.16	11.91 (< .001)

Values are presented as the mean ± standard deviation or n (%). IH = Intermediate hyperglycemia; FBS = Fasting blood sugar; HbA1c = Hemoglobin A1c.

**Table 2.** BMI, AO Markers, Hs-CRP, and Leptin-related Characteristics in the IH and Non-IH Groups (N = 248)

Variables/Categories	Total	IH group	Non-IH group	t/χ^2^ (*p*)
		FBS: 100-125 mg/dL or HbA1c: 5.7%-6.4%	FBS < 100 mg/dL or HbA1c < 5.7%	
		n = 130 (52.4%)	n = 118 (47.6%)	
BMI (kg/m^2^)	28.51 ± 4.90	29.89 ± 5.11	26.99 ± 4.19	4.17 (.042)
Normal weight (< 23.0)	34 (13.7)	12 (9.2)	22 (18.6)	16.63 (.001)
Overweight (23.0-24.9)	28 (11.3)	10 (7.7)	18 (15.3)	
Obesity (25.0-29.9)	100 (40.3)	49 (37.7)	51 (43.2)	
Severe obesity (≥ 30.0)	86 (34.7)	59 (45.4)	27 (22.9)	
WC (cm)	92.62 ± 13.89	97.26 ± 13.30	87.50 ± 12.73	5.89 (< .001)
Non-AO (male < 90 cm, female < 85 cm)	84 (33.9)	24 (18.5)	60 (50.8)	28.96 (< .001)
AO (male ≥ 90 cm, female ≥ 85 cm)	164 (66.1)	106 (81.5)	58 (49.2)	
Hs-CRP (mg/L)	1.52 ± 0.57	1.83 ± 1.67	1.17 ± 1.39	3.37 (.001)
< 1	137 (55.3)	60 (46.2)	77 (65.3)	
1-3	74 (29.8)	44 (33.8)	30 (25.4)	10.28 (.006)
> 3	37 (14.9)	26 (20.0)	11 (9.3)	
Leptin (ng/mL)	12.72 ± 8.99	14.51 ± 9.32	10.75 ± 8.20	3.35 (.001)

Values are presented as the mean ± standard deviation or n (%). IH = Intermediate hyperglycemia; FBS = Fasting blood sugar; HbA1c = Hemoglobin A1c; BMI = Body mass index; WC = Waist circumference; AO = Abdominal obesity; Hs-CRP = High sensitivity C-reactive protein.

**Table 3.** Correlations among BMI, WC, hs-CRP, and Leptin (N = 248)

Variables	BMI	WC	Hs-CRP	Leptin
	r (*p*)			
WC	.79 (< .001)	1	-	-
Hs-CRP	.49 (< .001)	.41 (< .001)	1	-
Leptin	.55 (< .001)	.37 (< .001)	.46 (< .001)	1

BMI = Body mass index; WC = Waist circumference; Hs-CRP = High sensitivity C-reactive protein.

**Table 4.** Predictive Variables for Increased IH (N = 248)

Variables	Categories	AOR	95% CI	*p*
BMI (ref. < 23.0 kg/m^2^)				
	Overweight (23.0-24.9)	0.98	0.43~2.21	.964
	Obesity (25.0-29.9)	3.52	0.52~23.71	.195
	Severe obesity (≥ 30.0)	4.01	0.66~30.95	.124
WC (ref. = male < 90 cm, female < 85 cm)				
	Male ≥ 90, female ≥ 85	5.45	2.09~12.44	< .001
Hs-CRP (ref. < 1 mg/L)				
	1-3	1.20	0.84~4.82	.102
	> 3	2.43	1.02~6.79	.004
Leptin		3.05	1.15~10.09	.003
Age (ref. = 19-29 years)				
	30-39	8.07	2.31~28.14	.001
	40-49	8.79	2.46~31.42	.001
	50-59	18.42	4.97~68.26	< .001
	≥ 60	35.33	7.82~159.64	< .001
Education level (ref. = high school)				
	Above college	4.01	0.68~9.97	.105
Economic level (ref. = low)				
	Medium	0.91	0.33~2.51	.861
	High	1.49	0.73~3.06	.267

IH = Intermediate hyperglycemia; AOR = Adjusted odds ratio; CI = Confidence interval; BMI = Body mass index; WC = Waist circumference; Hs-CRP = High sensitivity C-reactive protein; ref. = reference.

## Data Availability

Please contact the corresponding author for data availability.
